# Reduced Heart Function Predicts Drug-Taking Compliance and Two-Year Prognosis in Chinese Patients With Stable Premature Coronary Artery Disease

**DOI:** 10.14740/jocmr2045w

**Published:** 2014-12-29

**Authors:** Zhong Chen, Zhen Ding, Xin Wang, Xiaofeng Zhang, Genshan Ma

**Affiliations:** a Department of Cardiology, Shanghai Jiao Tong University Affiliated Sixth People’s Hospital, No. 600 Yishan Road, Shanghai 200233,China; b Department of Cardiology, Zhenjiang First People’s Hospital, Zhenjiang 212002, China; c Department of Cardiology, The Affiliated Zhongda Hospital of Southeast University, No. 87 Dingjiaqiao, Nanjing 210009, China; d Department of Cardiology, The Affiliated Nanjing Second Hospital of Southeast University, Nanjing 210009, China

**Keywords:** Atherosclerosis, Premature, Left ventricular ejection fraction, Drug-taking compliance, Major adverse cardiac events

## Abstract

**Background:**

The purpose of this study was to determine the association between heart function, compliance with drug administration, and the mid-term prognosis in Chinese patients with stable premature coronary artery disease (CAD) (male < 55 years and female < 65 years).

**Methods:**

The study included 512 patients with stable premature CAD. An estimated glomerular filtration rate (eGFR) calculated using the MDRD formula, baseline clinical characteristics, use of medications for coronary secondary prevention therapies (aspirin, β-blocker, angiotensin-converting enzyme inhibitors (ACEIs) or angiotensin receptor blockers, or statins), and 2-year follow-up results, in particular major adverse cardiac events (MACEs), were collected and analyzed.

**Results:**

Patients with reduced left ventricular ejection fraction (LVEF) (18.75%) were more prevalent among men, smokers, those with type 2 diabetes, with a family history of cardiovascular disease (CVD), and with higher white blood cells counts ((8.88 ± 0.35) × 10^9^/L vs. (6.90 ± 0.17) × 10^9^/L) (all P < 0.05) compared to those with preserved LVEF. There was no significant difference between creatinine or eGFR values in the two groups with reduced and preserved LVEF (all P > 0.05). Patients with LVEF < 50% in the MACEs group had a lower ratio of optimal drug administration compared to the MACEs-free group (Z = -0.228, P = 0.820 and Z = -2.167, P = 0.03 respectively). Patients with reduced LVEF had a significantly higher ratio of composite MACEs than patients with preserved LVEF during 2-year follow-up (47.13% vs. 33.50%, P < 0.05).

**Conclusions:**

Stable premature CAD patients with reduced LVEF have more risk factors, lower medication compliance, and worse 2-year outcomes than those with preserved LVEF.

## Introduction

Cardiovascular diseases (CVDs), including coronary artery disease (CAD), stroke, and peripheral vascular disease, are the leading causes of death worldwide. Patients with premature CAD (males < 55 years and females < 65 years) belong to a special subgroup, and the incidence of CVD is expected to increase in the following few decades in China. These patients with risk factors, such as obesity, cigarette smoking, type 2 diabetes mellitus (T2DM), hyperlipidemia, and family history of CVD, are at a higher risk for future cardiac death and worse outcomes including recurrent angina, recurrent myocardial infarction (MI), target vessel revascularization (TVR), and heart failure. Moreover, the impact of premature CAD on families is devastating [1].

Recently, significant advances in the treatment options for CAD have occurred, mainly related to the availability and implementation of evidence-based guidelines. Clinical trials of primary prevention have shown that antihypertensive therapy is associated with a 30-40% reduction in the incidence of cerebrovascular diseases within just a few years. Secondary prevention programs could improve the process of care, reduce re-admission to hospitals, and enhance the quality of life or functional status of patients with CAD and/or heart failure [2]. Guidelines [3, 4] have been developed and regularly updated to ensure wider coverage for all eligible patients to receive optimal treatment and for secondary prevention.

However, use of drugs including aspirin, β-blockers, angiotensin-converting enzyme inhibitors (ACEIs)/angiotensin II receptor blockers (ARBs), and statins remains suboptimal for secondary prevention in most countries [5-8]. A survey conducted in 22 European countries revealed that the use of cardioprotective medications among CAD patients was 78-91% for β-blockers, antiplatelets, and ACEIs/ARBs [5]. These data are similar to those collected in the United States [6]. Available data from India show that aspirin is prescribed in 91% of patients, compared to β-blockers in 69%, ACEIs/ARBs in 82%, and statins in 69% of patients [7]. In China, patients with acute coronary syndrome (ACS) without heart failure had higher prescription rates than in Europe and India [8].

In actual practice, many CAD patients have reduced left ventricular ejection fraction (LVEF), which is an independent risk indicator for a worse prognosis and higher risk of future cardiac events. However, until today there remain minimal data on the use of guidelines for recommending drugs for secondary prevention among younger patients in China. Thus, our study was designed to assess the real status of the association between heart function (expressed by LVEF) and the adherence to secondary preventive medications and 2-year prognosis in patients with stable premature CAD in a Chinese population.

## Methods

### Study population

From January 2005 to May 2009, 512 patients with a confirmed diagnosis of stable premature CAD (men < 55 years of age and women < 65 years of age) were consecutively enrolled from Jiangsu province, China. All patients underwent elective coronary angiography for the evaluation of coronary stenosis, and patients with ≥ 50% stenosis in at least one main coronary artery were considered to have CAD. Patients with ACS, congenital heart disease, syndrome X, or severe liver or kidney disease were excluded from the study. The investigation conforms to the principles outlined in the Declaration of Helsinki. The Medical Ethics Committee of the Affiliated Zhongda Hospital of Southeast University approved the study protocol, and written informed consent was obtained from all participants.

### Determination of risk factors and data collection

A questionnaire was completed according to the medical records of all participants including CAD risk factors like hypertension, T2DM, family history of CVD, and smoking status. Anthropometric measurements and blood pressure were determined according to standard protocols. Definition of hypertension, T2DM, smoking, and calculation of body mass index (BMI) have been previously described [9]. Two-dimensional echocardiography was performed to calculate LVEF according to American College of Cardiology and American Heart Association guidelines [10].

Plasma concentrations of total cholesterol (TC), triglycerides (TGs), high-density lipoprotein cholesterol (HDL-C), low-density lipoprotein cholesterol (LDL-C), apolipoprotein A1 (apoA1), apolipoprotein B (apoB), creatinine (Cr), white blood cell (WBC) counts, and hemoglobin (Hb) were determined using standard methods with an automatic chemical analyzer (Beckman Coulter Synchron Clinical System LX20, Fullerton, CA, USA). TG was measured directly when the results were > 4.52 mmol/L (400 mg/dL). An estimated glomerular filtration rate (eGFR) was calculated using the MDRD formula [11].

All patients were followed up from discharge to their first cardiac event or the end of the study period (April 30, 2011). The major follow-up issues were compliance with drug administration and major adverse cardiac events (MACEs), including recurrent angina, recurrent MI, TVR, re-admission for deteriorated heart failure and cardiac death. Treatment compliance was defined as taking the same category of drugs at discharge during the follow-up period.

### Statistical analyses

Statistical analyses were done using the SPSS 15.0 software (SPSS, Inc., Chicago, IL, USA). Continuous variables are expressed as mean ± SD, and the Student’s *t*-test or Chi-square test was used to analyze differences between the two study groups. Descriptive data are expressed as numbers and percentages. The significance of inter-group differences was analyzed using the Chi-square test. Trends in difference levels of types of drugs taken were assessed using the Wilcoxon rank sum test. Two-tailed P values < 0.05 were considered statistically significant.

## Results

### Baseline characteristics

The patient cohort consisted of 512 patients (mean age, 52.29 ± 10.17) and 210 (41.02%) of them were male. Ninety-six (18.75%) patients had reduced LVEF (< 50%), and 416 (81.25%) had preserved LVEF (≥ 50%). Compared to patients with preserved LVEF, patients with reduced LVEF were more likely to be male and have a higher ratio for family history of CVD, smoking, T2DM, multi-vessel disease, higher levels of fasting blood sugar (FBS) and WBCs (all P < 0.05). However, no significant differences existed between the ratio of hypertension and mean values of age, BMI, TG, LDL-C, HDL-C, apoA1, apoB, Cr, eGFR, and Hb between the two groups (all P > 0.05) (Table 1).

**Table 1:** Baseline Characteristics of Study Population

Characteristics	LVEF < 50% (n = 96)	LVEF ≥ 50% (n = 416)	P value
Age, years	51.98 ± 8.72	52.37 ± 8.33	0.605
BMI, kg/m^2^	25.20 ± 3.10	25.74 ± 2.87	0.113
Male, n (%)	63 (65.63)	147 (35.34)	0.000
Smoking, n (%)	34 (35.42)	101 (24.27)	0.026
Hypertension, n (%)	65 (67.71)	283 (68.03)	0.952
Type 2 diabetes mellitus, n (%)	25 (26.04)	71 (17.07)	0.042
Family history of CVD, n (%)	25 (26.04)	69 (16.59)	0.031
Single-vessel disease, n (%)	44 (45.83)	238 (57.21)	0.043
Multi-vessel disease, n (%)	52 (54.17)	178 (42.79)	0.043
Creatinine, µmol/L	87.33 ± 9.55	78.54 ± 2.83	0.239
eGFR, mL/min/1.73 m^2^	55.17 ± 14.77	57.44 ± 13.81	0.152
LDL-C, mmol/L	3.02 ± 0.11	2.92 ± 0.05	0.401
HDL-C, mmol/L	1.09 ± 0.04	1.15 ± 0.02	0.219
TG, mmol/L	1.56 ± 0.10	1.61 ± 0.06	0.710
apoA1, g/L	1.14 ± 0.09	1.11 ± 0.03	0.748
apoB, g/L	0.88 ± 0.03	0.84 ± 0.01	0.275
WBCs, × 10^9^/L	8.88 ± 0.35	6.90 ± 0.17	0.000
Hemoglobin, g/L	136.71 ± 1.83	138.43 ± 0.83	0.376
FBS, mmol/L	6.65 ± 0.27	5.54 ± 0.09	0.002

Data are expressed as the number of individuals (percentage in parentheses) or the mean ± SD, as appropriate. apoA1: apolipoprotein A1; apoB: apolipoprotein B; BMI: body mass index; CAD: coronary artery disease; CVD: cardiovascular disease; eGFR: estimated glomerular filtration rate; FBS: fasting blood sugar; HDL-C: high-density lipoprotein cholesterol; LDL-C: low-density lipoprotein cholesterol; LVEF: left ventricular ejection fraction; MI: myocardial infarction; TG: triglyceride; WBCs: white blood cells.

### Drug treatment for secondary cardiovascular prevention

The use of drugs for secondary cardiovascular prevention is displayed in Table 2. Analyses revealed that there was a gradual decrease in prescriptions for aspirin, β-blockers, ACEIs/ARBs, and statins during the follow-up period compared to prescriptions at hospital discharge. For in-hospital, 1- and 2-year follow-up, there was no significant difference in the use of drugs between the two groups. Meanwhile, compared to baseline data, the use of aspirin, β-blockers, and statins at 1-year and the use of β-blockers and statins at 2-year follow-up were lower within the preserved LVEF group (P < 0.05) (Table 2).

**Table 2:** Data on Drugs for Secondary Prevention at Baseline and During Follow-Up

	LVEF < 50% (n† = 96/88/87)	LVEF ≥ 50% (n† = 416/393/391)	P value
Aspirin			
In-hospital, 512	92 (95.83%)	402 (96.63%)	0.710
1-year, 481	83 (94.32%)	368* (93.64%)	0.812
2-year, 478	82 (94.25%)	367 (93.86%)	0.890
β-blockers			
In-hospital, 512	83 (86.46%)	365 (87.74%)	0.723
1-year, 481	73 (82.95%)	323* (82.19%)	0.865
2-year, 478	71 (81.61%)	321* (82.10%)	0.915
ACEIs/ARBs			
In-hospital, 512	70 (72.92%)	301 (72.36%)	0.912
1-year, 481	66 (75.00%)	289 (73.54%)	0.778
2-year, 478	63 (72.41%)	285 (72.89%)	0.928
Statins			
In-hospital, 512	85 (88.54%)	371 (89.18%)	0.856
1-year, 481	74 (84.09%)	332* (84.47%)	0.928
2-year, 478	70 (80.46%)	317* (81.07%)	0.895

*P < 0.05, compared with in-hospital data within the LVEF ≥ 50% group. †The available number of patients in-hospital, and at 1- and 2-year follow-up. ACEIs: angiotensin-converting enzyme inhibitors; ARBs: angiotensin II receptor blockers; LVEF: left ventricular ejection fraction.

### Relationship between LVEF, follow-up duration, and MACEs

During 1- and 2-year follow-up, there was no significant difference among any single MACEs between the two groups. However, compared to those with preserved LVEF, patients with low LVEF had a higher ratio of composite MACEs during 2-year follow-up (47.13% vs. 33.50%, P < 0.05) (Table 3).

**Table 3:** Follow-Up Results of Cardiovascular Events

	LVEF < 50% (n† = 88/87)	LVEF ≥ 50% (n† = 393/391)	P value
Recurrent angina			
1-year	11 (12.50%)	46 (11.70%)	0.979
2-year	14 (16.09%)	56 (14.32%)	0.799
Readmission for heart failure			
1-year	3 (3.41%)	7 (1.78%)	0.333
2-year	5 (5.75%)	9 (2.30%)	0.085
Recurrent MI			
1-year	2 (2.27%)	5 (1.27%)	0.479
2-year	4 (4.59%)	8 (2.05%)	0.169
TVR			
1-year	13 (14.77%)	41 (10.43%)	0.244
2-year	17 (19.54%)	56 (14.32%)	0.221
Cardiac death			
1-year	1 (1.14%)	2 (0.05%)	0.245
2-year	1 (1.15%)	2 (0.05%)	0.243
Composite MACEs			
1-year	30 (34.09%)	101 (25.70%)	0.084
2-year	41 (47.13%)	131 (33.50)	0.017

†The available number of patients during 1- and 2-year follow-up. LVEF: left ventricular ejection fraction; MACEs: major adverse cardiac events; MI: myocardial infarction; TVR: target vessel revascularization.

### Relationship between drug-taking adherence and composite MACEs

When patients were divided into four groups according to the numbers for secondary preventive drugs consumed (aspirin, β-blockers, ACEIs/ARBs, and statins), the 2-year follow-up results showed patients with LVEF < 50% had a lower ratio of optimal drug administration in the MACEs group compared to the MACEs-free group (Z = -0.228, P = 0.820) (Table 4).

**Table 4:** Relationship Between Drug Usage and Composite MACEs After 2-Year Follow-Up

	LVEF < 50% (n = 87)		LVEF ≥ 50% (n = 391)	
	MACEs (n = 41)	Without MACEs (n = 46)	MACEs (n = 131)	Without MACEs (n = 260)
Any one drug treatment	0	2	2	4
Any two drugs treatment	9	8	27	31
Any three drugs treatment	15	18	58	113
All four drugs treatment	17	18	44	111
P value	Z = -0.228, P = 0.820		Z = -2.167, P = 0.03	

LVEF: left ventricular ejection fraction; MACEs: major adverse cardiac events.

## Discussion

The present study proved that stable premature CAD patients with reduced LVEF have more risk factors, lower compliance to drug administration, and worse 2-year outcomes than those with preserved LVEF.

Recently, the benefits of optimal medical care on the risks of MACEs have been demonstrated in clinical trials. Patients with prior MIs had a 43% increased risk of heart failure, and patients with lower LVEF values (41-50%) had a 41% increased risk [12]. Even in young adults with an average LVEF (55-57%), every 10% drop in LVEF values predicted a substantial increase in mortality [13].

In our study, patients with reduced LVEF were more likely to be male, had a higher prevalence of family history of CVD, smoking, T2DM, multi-vessel disease, higher levels of FBS, and WBCs counts. Moreover, these patients had more composite MACEs during 2-year follow-up, suggesting that their mid-term prognosis was poor. Therefore, more effective measures should be taken to control such modifiable risk factors as smoking, T2DM, and hypertension among stable premature CAD patients with low LVEF, including optimal medication usage and life-style interventions. Patients at risk for heart failure with LVEF ≤ 40% can make changes in their lifestyle to achieve similar medical and psychosocial benefit to those of patients with normal LVEF [14]. Also, a recent study showed that CAD patients with asymptomatic reduced LVEF could safely delay revascularization by lifestyle modification without increased risk for cardiac events or overt heart failure for 3 years [15].

Reduced kidney function is a risk factor for CVD development and progression as reported in several studies, as well as in populations at high risk for CAD, and according to updated guidelines [16]. In the ARIC study, patients with eGFR < 60 mL/min/1.73 m^2^ were at a high risk for developing heart failure no matter whether they had prevalent CAD or not [17]. The same results were demonstrated in the PEACE study [12]. Our primary study results show that serum uric acid level is negatively associated with renal function, as assessed by eGFR, and serves as an independent predictor for chronic kidney disease in patients with stable CAD and T2DM [18], and chronic kidney disease predicts poor prognosis in patients with stable premature CAD [9]. However, in the present study, mean eGFR was (55.17 ± 14.77) mL/min/1.73 m^2^ in the LVEF-decreased group and (57.44 ± 13.81) mL/min/1.73 m^2^ in the LVEF-preserved group, and no significant difference existed between the two groups, which suggests that the value of renal function assessment using eGFR might decrease among this specific cohort.

Guidelines based on evidence from randomized controlled trails recommend that aspirin, β-blockers, ACEIs/ARBs, and statins be used in all patients with or at risk of developing CAD [19]. It has been the consensus that, if used optimally, these agents could reduce long-term risks of cardiovascular events and mortality. Yet, use of secondary prevention therapies remains low according to reports in the available literature [5-8]. Compared with the data described above, our study revealed a remarkably higher ratio of drug administration in patients with stable premature CAD in our study population. This may represent more appropriate use of evidence-based therapies at tertiary care clinics, which differs from that in primary and secondary care units. However, administration of these drugs declined, with LVEF < 50% patients suffering from more cardiovascular events during the 2-year follow-up. Thus, evidence-proven drugs should be proposed as a means of improving cardiovascular prevention to reduce adverse cardiovascular outcomes by increasing patient adherence to these optimal therapies.

Some strengths and limitations of this study should be mentioned. First, this was a single-center study involving subjects with documented stable premature CAD patients, so it is unlikely that the subjects adequately represented population characteristics included in other studies [5-8]. However, our study sample is similar to that routinely seen by practitioners. Second, we did not have access to any information regarding patients’ lifestyles, such as physical inactivity and obesity, which are well-documented risk factors for CAD and might be associated with patients’ adherence to drug therapy. However, these patients underwent regular visits to our outpatient office. This exists in both groups and is less likely to influence the comparison between the two groups. Finally, our study sample is small and only reports mid-term outcomes; a larger study sample and long-term follow-up is needed to acquire more convincing data in this specific field.

Based on the study findings, the real-world drug use patterns and follow-up results in the Chinese setting show that young stable CAD patients with reduced LVEF have more cardiovascular risk factors, lower drug administration compliance, and worse 2-year prognosis. Educational strategies to increase awareness of the up-dated guidelines [20] among physicians and nurses must be strengthened. In addition, exploring more useful educational programs and methods [21, 22] to improve patients’ adherence to optimal drug therapy is also needed.

## References

[1] Klein LW, Nathan S (2003). Coronary artery disease in young adults. J Am Coll Cardiol.

[2] Khunti K, Stone M, Paul S, Baines J, Gisborne L, Farooqi A, Luan X (2007). Disease management programme for secondary prevention of coronary heart disease and heart failure in primary care: a cluster randomised controlled trial. Heart.

[3] Smith SC, Jr., Allen J, Blair SN, Bonow RO, Brass LM, Fonarow GC, Grundy SM (2006). AHA/ACC guidelines for secondary prevention for patients with coronary and other atherosclerotic vascular disease: 2006 update endorsed by the National Heart, Lung, and Blood Institute. J Am Coll Cardiol.

[4] Graham I, Atar D, Borch-Johnsen K, Boysen G, Burell G, Cifkova R, Dallongeville J (2007). European guidelines on cardiovascular disease prevention in clinical practice: executive summary. Atherosclerosis.

[5] Kotseva K, Wood D, De Backer G, De Bacquer D, Pyorala K, Keil U (2009). EUROASPIRE III: a survey on the lifestyle, risk factors and use of cardioprotective drug therapies in coronary patients from 22 European countries. Eur J Cardiovasc Prev Rehabil.

[6] Lewis SJ, Robinson JG, Fox KM, Grandy S (2010). Underutilisation of cardiovascular medications among at-risk individuals. Int J Clin Pract.

[7] Sharma KK, Gupta R, Agrawal A, Roy S, Kasliwal A, Bana A, Tongia RK (2009). Low use of statins and other coronary secondary prevention therapies in primary and secondary care in India. Vasc Health Risk Manag.

[8] Wang N, Zhao D, Liu J, Yu CM, Wang W, Sun J, Li Y (2012). Impact of heart failure on in-hospital outcomes of acute coronary syndrome patients in China - results from the Bridging the Gap on CHD Secondary Prevention in China (BRIG) project. Int J Cardiol.

[9] Ding Z, Wang X, Chen Z, Zhang X, Tang C, Feng Y, Ma G (2012). Chronic kidney disease predicts poor prognosis in patients with stable premature coronary artery disease. Eur J Intern Med.

[10] Cheitlin MD, Armstrong WF, Aurigemma GP, Beller GA, Bierman FZ, Davis JL, Douglas PS (2003). ACC/AHA/ASE 2003 guideline update for the clinical application of echocardiography: summary article: a report of the American College of Cardiology/American Heart Association Task Force on Practice Guidelines (ACC/AHA/ASE Committee to Update the 1997 Guidelines for the Clinical Application of Echocardiography). Circulation.

[11] Levey AS, Bosch JP, Lewis JB, Greene T, Rogers N, Roth D (1999). A more accurate method to estimate glomerular filtration rate from serum creatinine: a new prediction equation. Modification of Diet in Renal Disease Study Group. Ann Intern Med.

[12] Lewis EF, Solomon SD, Jablonski KA, Rice MM, Clemenza F, Hsia J, Maggioni AP (2009). Predictors of heart failure in patients with stable coronary artery disease: a PEACE study. Circ Heart Fail.

[13] Cole JH, Miller JI, 3rd, Sperling LS, Weintraub WS (2003). Long-term follow-up of coronary artery disease presenting in young adults. J Am Coll Cardiol.

[14] Pischke CR, Weidner G, Elliott-Eller M, Ornish D (2007). Lifestyle changes and clinical profile in coronary heart disease patients with an ejection fraction of <or=40% or >40% in the Multicenter Lifestyle Demonstration Project. Eur J Heart Fail.

[15] Pischke CR, Elliott-Eller M, Li M, Mendell N, Ornish D, Weidner G (2010). Clinical events in coronary heart disease patients with an ejection fraction of 40% or less: 3-year follow-up results. J Cardiovasc Nurs.

[16] Reiner Z, Catapano AL, De Backer G, Graham I, Taskinen MR, Wiklund O, Agewall S (2011). ESC/EAS Guidelines for the management of dyslipidaemias: the Task Force for the management of dyslipidaemias of the European Society of Cardiology (ESC) and the European Atherosclerosis Society (EAS). Eur Heart J.

[17] Kottgen A, Russell SD, Loehr LR, Crainiceanu CM, Rosamond WD, Chang PP, Chambless LE (2007). Reduced kidney function as a risk factor for incident heart failure: the atherosclerosis risk in communities (ARIC) study. J Am Soc Nephrol.

[18] Chen Z, Ding Z, Fu C, Yu C, Ma G (2014). Correlation between serum uric Acid and renal function in patients with stable coronary artery disease and type 2 diabetes. J Clin Med Res.

[19] Bairey Merz CN, Alberts MJ, Balady GJ, Ballantyne CM, Berra K, Black HR, Blumenthal RS (2009). ACCF/AHA/ACP 2009 competence and training statement: a curriculum on prevention of cardiovascular disease: a report of the American College of Cardiology Foundation/American Heart Association/American College of Physicians Task Force on Competence and Training (Writing Committee to Develop a Competence and Training Statement on Prevention of Cardiovascular Disease): developed in collaboration with the American Academy of Neurology; American Association of Cardiovascular and Pulmonary Rehabilitation; American College of Preventive Medicine; American College of Sports Medicine; American Diabetes Association; American Society of Hypertension; Association of Black Cardiologists; Centers for Disease Control and Prevention; National Heart, Lung, and Blood Institute; National Lipid Association; and Preventive Cardiovascular Nurses Association. Circulation.

[20] Fihn SD, Blankenship JC, Alexander KP, Bittl JA, Byrne JG, Fletcher BJ, Fonarow GC (2014). 2014 ACC/AHA/AATS/PCNA/SCAI/STS Focused Update of the Guideline for the Diagnosis and Management of Patients With Stable Ischemic Heart Disease: A Report of the American College of Cardiology/American Heart Association Task Force on Practice Guidelines, and the American Association for Thoracic Surgery, Preventive Cardiovascular Nurses Association, Society for Cardiovascular Angiography and Interventions, and Society of Thoracic Surgeons. J Am Coll Cardiol.

[21] Allen JK, Dennison CR (2010). Randomized trials of nursing interventions for secondary prevention in patients with coronary artery disease and heart failure: systematic review. J Cardiovasc Nurs.

[22] Soran OZ, Feldman AM, Pina IL, Lamas GA, Kelsey SF, Selzer F, Pilotte J (2010). Cost of medical services in older patients with heart failure: those receiving enhanced monitoring using a computer-based telephonic monitoring system compared with those in usual care: the Heart Failure Home Care trial. J Card Fail.

